# The Association between Body Mass Index and Leisure-Time Physical Activity in Adults with Multiple Sclerosis

**DOI:** 10.3390/ijerph17030920

**Published:** 2020-02-02

**Authors:** Wi-Young So, Alon Kalron

**Affiliations:** 1 Sports and Health Care Major, College of Humanities and Arts, Korea National University of Transportation, Chungju-si 27469, Korea; wowso@ut.ac.kr; 2 Department of Physical Therapy, School of Health Professions, Sackler Faculty of Medicine, Tel-Aviv University, Tel Aviv, Ramat Aviv 6997801, Israel; 3 Multiple Sclerosis Center, Sheba Medical Center, Tel-Hashomer, Ramat Gan 52621, Israel; 4 Sagol School of Neuroscience, Tel-Aviv University, Tel Aviv, Ramat Aviv 6997801, Israel

**Keywords:** body mass index, disability, leisure-time physical activity, multiple sclerosis, obesity

## Abstract

(1) Purpose: Conflicting information exists regarding the relationship between obesity, leisure-time physical activity (PA), and disability in people with multiple sclerosis (PwMS). We aimed to investigate the association between leisure-time PA and weight status in a relatively large cohort of PwMS. Furthermore, we examined this relationship according to the level of neurological disability. (2) Methods: The study included 238 PwMS (138 women) with a mean Expanded Disability Status Scale (EDSS) score of 2.5 (standard deviation [SD] = 1.7), mean disease duration of 6.4 (SD = 8.2) years, and mean age of 40.5 (SD = 12.9) years. Obesity was defined using two different metrics, each based on body mass index (BMI). Leisure-time PA was determined by the Godin–Shephard leisure-time PA questionnaire. Statistical analyses included multivariate logistic regression, the chi-square test, and Pearson coefficient correlations. (3) Results: The unadjusted odds ratio (OR) between leisure-time PA and BMI based on the World Health Organization’s (WHO) definition was 1.070 (*p* = 0.844) for overweight and 1.648 (*p* = 0.254) for obesity. The adjusted OR was 1.126 (*p* = 0.763) for overweight and 1.093 (*p* = 0.847) for obesity after adjustment for age, gender, and disability status. Chi-square analysis revealed no significant correlation between leisure-time PA and obesity (*p* = 0.564) according to the BMI threshold for PwMS. The unadjusted OR (95% confidence interval [CI]) between disability level and BMI based on the WHO definition was 1.674 (*p* = 0.220) for overweight and 0.618 (*p* = 0.460) for obesity. The adjusted OR was 1.130 (*p* = 0.787) for overweight and 0.447 (*p* = 0.234) for obesity after adjustment for age, gender, and leisure-time PA. Similarly, chi-square analysis revealed no significant correlation between disability level and obesity (*p* = 0.701) per the BMI threshold for PwMS. (4) Conclusions: No association was found between leisure-time PA and BMI in PwMS. An additional finding was the absence of any association between obesity and neurological disability level in the multiple sclerosis cohort.

## 1. Introduction

Multiple sclerosis (MS) is an autoimmune and demyelination disease of the central nervous system that causes progressive accumulation of disability over time [1]. Overweight and obesity typify common comorbid conditions found with MS. Marck et al. found that among 2399 people with MS (PwMS), 22.5% were overweight and 19.4% were obese. Notably, overweight and obese PwMS have higher rates of diabetes, elevated blood pressure, increased insulin resistance, blood lipid issues, depression, anxiety, and arthritis compared with people of normal weight [2].

Although physical activity (PA) is a standard recommendation for weight control in the general population [3], conflicting evidence exists regarding the relationship between body weight status and PA in PwMS. One study investigated the effects of a 6-month internet-delivered PA behavioral intervention on the body composition of 82 PwMS. The researchers reported that although the intervention resulted in higher intensities of PA, there were no significant differences in body composition [4]. A recent study reported that 12 weeks of high-intensity aerobic and strength training did not alter the total fat and lean body mass in the MS sample [5]. In contrast, a long-term (12 months) endurance exercise program resulted in a significant decrease in body fat in 89 PwMS individuals [6]. Moreover, 24 weeks of combined endurance and resistance training demonstrated improvements in body composition, especially in the lean tissue mass in a group of 22 PwMS [7]. This inconclusive evidence reinforces the need for new information in order to clarify the relationship between body weight and PA in the MS population.

In the present study, we focused on leisure-time PA (i.e., walking, dancing, hiking, and swimming) that people typically engage in during their free time. It is worth noting that the World Health Organization’s (WHO) global recommendations to perform PA to benefit one’s health are mainly based on leisure-time PA. Therefore, the primary aim of our study was to examine the relationship between weight status (via body mass index) and leisure-time PA in a cohort of PwMS. Furthermore, we examined this relationship according to the patient’s level of neurological disability, represented by the Expanded Disability Status Scale (EDSS) score. Our hypothesis was that obesity would be related to decreased participation in leisure time PA and a higher level of disability.

## 2. Method

### 2.1. Study Design and Participants

This was an observational cross-sectional study comprising 238 PwMS (138 women and 100 men) recruited from the Multiple Sclerosis Center, Sheba Medical Center, Tel-Hashomer, Israel. Inclusion criteria were as follows: (1) a neurologist-confirmed diagnosis of definite MS according to the revised McDonald criteria [8]; (2) an EDSS score < 7.0 [9] and consistently able to walk at least 20 meters without resting; and (3) completion of the Godin leisure-time exercise self-report questionnaire (GLTEQ) between January 2015 and July 2019. Exclusion criteria were as follows: (1) orthopedic disorders that could negatively affect physical activity performance, (2) pregnancy, (3) blurred vision, (4) cardiovascular and/or respiratory disorders, and (5) treatment with steroids due to relapse. The study was approved by the Sheba Medical Center Research Ethics Committee (Ethics Ref: 5596-08/244811), confirming extraction of demographical and clinical data for analysis and full exemption of written or verbal consent from the study participants.

### 2.2. Godin Leisure-Time Exercise Questionnaire (GLTEQ)

The GLTEQ, a self-administered measuring tool for assessing physical activity [10], is a valid self-report measure of PA in PwMS [11]. The questionnaire contains three items measuring the frequency of strenuous (e.g., jogging), moderate (e.g., fast walking), and mild (e.g., easy walking) exercise for periods > 15 minutes during the individual’s free time throughout a typical week. According to the instructions, the weekly frequencies of strenuous, moderate, and mild activities occurring over a 7-day period are multiplied by 9, 5, and 3 metabolic equivalents, respectively, and then summed to form a measure of total leisure activity. PwMS were divided into PA subgroups in accordance with Godin’s classification guidelines. A score ≥ 14 on the GLTEQ classified the patient as active (moderately, at least), and patients who scored < 14 were classified as insufficiently active [10]. According to a recent review, the GLTEQ is an appropriate and effective tool assisting in describing patterns of PA and examining correlates and outcomes of PA participation in the MS population [11].

### 2.3. Weight Status

Two different methods of assessing obesity were used: (i) based on the WHO standard of obesity, individuals with a BMI < 25 kg/m^2^, 25–30 kg/m^2^, and > 30 kg/m^2^ are classified as normal, overweight, and obese, respectively [12]; and (ii) values published by Pilutti and Motl (2016), who reported that the BMI threshold that best identifies obesity in PwMS is 24.7 kg/m^2^, are considered. Using this metric, patients with a BMI ≥ 24.7 kg/m^2^ were classified as obese and patients with a BMI < 24.7 kg/m^2^ were classified as normal [13]. A fixed stadiometer was used to measure height. Height was measured without shoes, with a small gap (10 cm) between the legs, feet pointed straight ahead, and ear canal level with the cheekbone. Weight was measured with a balanced beam scale. The participant was instructed to remove shoes and heavy outer garments, empty pockets, and stand still in the center of the platform.

### 2.4. Expanded Disability Status Scale (EDSS)

The EDSS, an accepted method of quantifying neurological disability in MS, consists of an eight-function system scale monitoring motor, sensory, cerebellar, brain stem, visual, bowel and bladder, pyramidal, and other functions. Each domain is graded from 0 = no disability to 5 or 6 = maximal disability [9]. According to the score achieved from each functional system, an integrated score between 0 (normal examination) and 10 (death) from MS is derived. A score ranging from 1.0 to 4.5 denotes patients who are fully ambulatory without aid; a score from 5.0 to 7.5 reveals moderate to severe impairment in ambulation. An EDSS level of 6.0 is primarily defined by the need for a unilateral aid for walking at least 100 m; an EDSS level of 6.5 is defined by the need for a bilateral walking aid; and a score from 8.0 to 9.5 refers to PwMS essentially restricted to bed.

### 2.5. Statistical Analysis

Descriptive statistics determined the demographic and clinical characteristics of the study participants. Outliers were ascertained for each outcome using box plots. Obesity values and GLTEQ scores were normally distributed according to the Kolmogorov-Smirnov test. In addition, PwMS were divided into two levels of disability based on their global EDSS score. An EDSS score ≤ 4.0 was defined as mildly disabled, and a score > 4.0 was defined as moderately-severely disabled. Multivariate logistic regression analyses were carried out to determine whether obesity was related to leisure-time PA and disability level, with and without adjusting for age and gender. Disability level was used as a covariate when assessing leisure-time PA and vice versa. A chi-squared analysis and a Pearson correlation were also performed. All analyses were performed using the SPSS software (Version 25.0 for Windows, IBM Corp., Armonk, NY, USA). All reported *p*-values were two-tailed, and the level of significance was set at *p* < 0.05.

## 3. Results

The median EDSS for the entire study group was 2.0 (range 0.0–6.5), mean disease duration was 6.4 (standard deviation [SD] = 8.2) years, and mean age was 40.5 (SD = 12.9) years. The mean BMI and GLTEQ scores of the total group were 24.7 (SD = 4.7) and 16.4 (SD = 18.0), respectively. Demographic and clinical characteristics according to weight groups are presented in Table 1. The odds ratio (OR) (95% confidence interval [CI]) between leisure-time PA and BMI according to the WHO definition was 1.070 (0.548–2.086; *p* = 0.844) for overweight and 1.648 (0.698–3.888; *p* = 0.254) for obesity without adjustment, and 1.126 (0.521–2.436; *p* = 0.763) for overweight and 1.093 (0.442–2.702; *p* = 0.847) for obesity after adjusting for age, gender, and disability status (Table 2). According to the chi-squared analysis, no correlation was found (*x^2^* = 0.420, *p* = 0.564) between leisure-time PA and obesity (based on the BMI threshold for PwMS provided by Pilutti and Motl [2016]) (Table 3).

**Figure 1:**
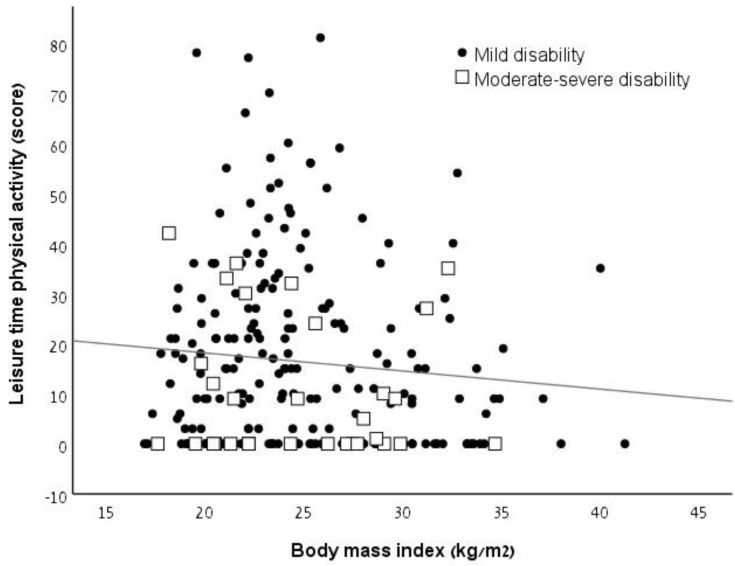
Relationship between obesity and leisure-time physical activity according to disability subgroups.

The OR (95% CI) between disability level and BMI according to WHO’s definition was 1.674 (0.735–3.810; *p* = 0.220) for overweight and 0.618 (0.172–2.221; *p* = 0.460) for obesity without adjustment, and 1.130 (0.466-2.738; *p* = 0.787) for overweight and 0.447 (0.119-1.682; *p* = 0.234) for obesity after adjustment for age, sex, and leisure-time PA (Table 4). The chi-squared analysis showed no relationship between level of disability and obesity (*x^2^* = 0.179, *p* = 0.701) according to the BMI thresholds for obesity presented by Pilutti and Motl (2016) (Table 5). Figure 1 illustrates the relationship between leisure-time PA and BMI according to the disability subgroups.

## 4. Discussion

Our main objective was to investigate the relationship between PA and obesity in PwMS. Accordingly, no associations were found between leisure-time PA and BMI in PwMS. This conclusion was confirmed by WHO’s criteria for obesity and the cutoff scores of Pilluti and Motl (2016) for assessing obesity in PwMS. Our outcome is in agreement with previous studies that did not demonstrate a relationship between PA with being overweight or obese, based on BMI [14,15,16]. Furthermore, the recent review of Ewanchuk et al. strengthens this line of evidence [17]. The authors found that 11 (out of 17) exercise interventional studies did not demonstrate any effect of PA programs (aerobic, resistance, and combined) on obesity outcomes, including BMI.

Nonetheless, there is evidence suggesting that PwMS who perform more PA have a lower BMI and are less likely to be obese [18,19]. This discrepancy may be explained by the parameters used to assess obesity. Although BMI is the most common measurement for obesity, it does have several limitations. Mainly, it does not distinguish between body fat, lean tissue mass, and bone mineral density. There is the possibility that including additional measures of body composition in the present study may have resulted in different conclusions.

Some may argue that the decision to focus solely on leisure-time PA may possibly be the reason that no significant associations were found with obesity. It is worth noting that according to the American College of Sports Medicine’s weight loss and controlling guidelines: (i) at least 150 min per week of moderate intense PA is needed to prevent weight gain; (ii) 150–250 min per week (approximately 1200–2000 kcal per week) of moderate intense PA is needed to prevent weight gain >3% and is associated with modest weight loss, and (iii) approximately 250–300 min per week (approximately 2000 kcal per week) of moderate intense PA is needed for greater weight loss and prevention of weight regain [3].

An additional finding of our study was the absence of any association between obesity and disability level in the MS cohort. Although this topic has been thoroughly investigated, the existing evidence is contradictory. Several previous studies have found that weight status is related to mobility and associated disability levels, indicating that PwMS who were less mobile (higher EDSS) had a higher BMI [20,21]. In contrast, Pilutti et al. (2012) reported that over a 24-month time period, BMI was not predictive of disability in a sample of 269 people with relapsing-remitting MS [14]. In a study of mild-moderate PwMS (median EDSS = 4.0) [21], an association between disability and weight status was not identified. Collectively, the research does not support a strong and consistent association between disability and BMI in PwMS, and our data strengthen this line of evidence. Furthermore, although not directly connected with disability, it is worth noting that the risk of cardiovascular disease, which is increased in PwMS, is not related to obesity or changes in body composition [22]. Therefore, we believe that BMI is not as important of a factor in disability as other demographic and clinical characteristics in PwMS. Moreover, it is questionable if BMI should be targeted as a means for reducing disability in MS.

Regardless of our results, we find it important to notice that the benefits of PA in PwMS have been well documented [23]. PA beneficially affects a variety of MS symptoms (i.e., fatigue, mobility, depressive symptoms, muscle strength, and quality of life). Moreover, recent evidence suggests that exercise may also have disease-modifying effects and possibly preventive effects by lowering disease risk.

The main strengths of this study include the relatively large sample and the use of two criteria for obesity. Nevertheless, our study has several limitations that warrant attention. First, dietary patterns, smoking, and alcohol consumption, all of which affect weight status, were not examined. Second, leisure time PA was quantified solely by a self-report questionnaire and not by direct measurement of the participants’ PA levels. Although the GLTEQ is a recommended measuring tool for evaluating PA in PwMS [11], it does have certain disadvantages. One disadvantage of this tool is that it does not take into account the length of time it takes to perform the PA, only that it lasts for at least 15 consecutive minutes. For instance, there was no difference in the score between a person who performed fast walking 3 times a week for 20 minutes or 3 times a week for one hour. Therefore, although the GLTEQ is recommended to differentiate between active and insufficiently active people, there is a possibility that it lacks the sensitivity to differentiate between the activity levels recommended for weight control. We speculate that PA assessed with other tools such as accelerometers and pedometers might demonstrate different relationships with BMI compared with leisure-time PA in the MS population. Additionally, there seems to be a discrepancy between objectively vs. subjectively assessed PA in PwMS [24]. Moreover, in a recent validation study of the GLTEQ, it was found that the GLTEQ scores primarily reflect moderate-to-vigorous PA rather than light PA and sedentary behavior in PwMS [25]. Finally, this was a cross-sectional study; consequently, we cannot determine causality (although no relationships were demonstrated).

## 5. Conclusions

The relatively high prevalence of obesity and overweight among PwMS, together with the importance of PA in this population, was the main incentive of this study. We sought to examine the relationship between these factors in relation to the level of disability. Although no relationship was found between BMI and PA or disability, we encourage future research to examine these relationships using various assessment tools in order to improve management of the MS population.

## Figures and Tables

**Table 1:** Demographic and clinical characteristics of the study sample (n = 238).

Variables	World Health Organization Standard of Obesity				Obesity as Defined for People with MS		
	Normal (n = 144)	Overweight (n = 57)	Obese (n = 37)	*p*-Value	Normal (n = 142)	Obese (n = 96)	*p*-Value
Age (years)	38.07 ± 12.15	46.18 ± 13.04	40.93 ± 13.09	<0.001 *	38.06 ± 12.23	43.99 ± 13.14	<0.001 *
Sex (female/male)	87/57	31/26	20/17		86/56	52/44	
Height (cm)	169.14 ± 9.08	168.32 ± 9.15	167.43 ± 10.29	0.576	169.11 ± 8.99	168.03 ± 9.70	0.379
Weight (kg)	62.04 ± 9.35	77.31 ± 10.13	92.86 ± 12.39	<0.001 *	61.88 ± 9.21	83.21 ± 13.46	<0.001 *
BMI (kg/m^2^)	21.60 ± 1.99	27.18 ± 1.38	33.06 ± 2.64	<0.001 *	21.55 ± 1.97	29.39 ± 3.52	<0.001 *
Disease duration (years)	5.52 ± 8.03	9.31 ± 9.06	5.38 ± 6.86	0.020 *	5.52 ± 8.03	7.69 ± 8.41	0.068
EDSS (score)	2.53 ± 1.73	2.82 ± 1.65	2.70 ± 1.58	0.573	2.54 ± 1.74	2.76 ± 1.60	0.341
Pyramidal	1.37 ± 1.23	1.56 ± 1.29	1.58 ± 1.20	0.518	1.37 ± 1.23	1.56 ± 1.24	0.274
Cerebellar	0.74 ± 0.97	0.67 ± 0.92	0.73 ± 0.91	0.917	0.74 ± 0.97	0.69 ± 0.91	0.663
Sensory	0.79 ± 1.05	0.87 ± 0.97	0.85 ± 1.00	0.895	0.78 ± 1.05	0.87 ± 0.98	0.531
GLTEQ (score)	18.18 ± 18.16	15.26 ± 19.27	11.43 ± 13.96	0.106	18.16 ± 18.14	13.88 ± 17.47	0.071

Data are expressed as mean ± standard deviation. * *p* < 0.05; tested by one-way analysis of variance or independent *t*-test.; MS: multiple sclerosis; BMI: body mass index; EDSS: expanded disability status scale; GLTEQ: Godin leisure-time exercise questionnaire.

**Table 2:** Physical activity in relation to World Health Organization’s standard of obesity assessed by multivariable logistic regression analysis.

BMI	Active vs. Insufficiently Active					
	n	ß	SE	OR	95% CI	*p*-Value
Model 1 (without adjustment for age, sex, and disability status)						
Normal	144	Reference		1.000		
Overweight	57	0.067	0.341	1.070	0.548–2.086	0.844
Obesity	37	0.499	0.438	1.648	0.698–3.888	0.254
Model 2 (with adjustment for age, sex, and disability status)						
Normal	144	Reference		1.000		
Overweight	57	0.119	0.394	1.126	0.521–2.436	0.763
Obesity	37	0.089	0.462	1.093	0.442–2.702	0.847

BMI: body mass index; SE: standard error; OR: odds ratio; CI: confidence interval; Normal: BMI <25 kg/m^2^; overweight: 25-30 kg/m^2^; obese: ≥30 kg/m^2^ (WHO’s standard of obesity, 1995); Active: scores ≥24 points; insufficiently active <24 points (Amireault and Godin, 2015).

**Table 3:** Physical activity in relation to obesity assessed by chi-squared analysis.

BMI	Active	Insufficiently Active	Total	*x^2^*	*p*-Value
Normal	44 (31.0%)	98 (69.0%)	142 (100.0%)	0.420	0.564
Obesity	26 (27.1%)	70 (72.9%)	96 (100.0%)		
Total	70 (29.4%)	168 (70.6%)	238 (100.0%)		

BMI: body mass index; Normal: BMI <24.7 kg/m^2^; obesity: ≥24.7 kg/m^2^ (Pilutti and Motl, 2016). Active: ≥24 points; insufficiently active: <24 points (Amireault and Godin, 2015).

**Table 4:** Disability status in relation to World Health Organization’s standard of obesity assessed by multivariable logistic regression analysis.

BMI	Mild vs. Moderate-Severe					
	n	ß	SE	OR	95% CI	*p*-Value
Model 1 (without adjustment for age, sex, and physical activity)						
Normal	144	Reference		1.000		
Overweight	57	0.515	0.420	1.674	0.735–3.810	0.220
Obesity	37	−0.482	0.653	0.618	0.172–2.221	0.460
Model 2 (with adjustment for age, sex, and physical activity)						
Normal	144	Reference		1.000		
Overweight	57	0.122	0.452	1.130	0.466–2.738	0.787
Obesity	37	−0.806	0.676	0.447	0.119–1.682	0.234

BMI: body mass index; SE: standard error; OR: odd ratio; CI: confidence interval. Normal: BMI <25 kg/m^2^; overweight: 25–30 kg/m^2^; obese: ≥30 kg/m^2^ (WHO’s standard of obesity, 1995). Mildly disabled: EDSS ≤4.0 points; Moderately-severely disabled >4.0 points by Kurtzke (1983).

**Table 5:** Disability status in relation to obesity assessed by chi-squared analysis.

BMI	Mild Disability	Moderate-Severe Disability	Total	*x^2^*	*p*-Value
Normal	124 (87.3%)	18 (12.7%)	142 (100.0%)	0.179	0.701
Obesity	82 (85.4%)	14 (14.6%)	96 (100.0%)		
Total	206 (86.6%)	32 (13.4%)	238 (100.0%)		

BMI: body mass index; Normal: BMI <24.7 kg/m^2^; obesity: ≥24.7 kg/m^2^ (Pilutti and Motl, 2016). Mildly disabled: Expanded Disability Status Scale ≤4.0 points; Moderately-severely disabled >4.0 points (Kurtzke, 1983).
